# Maternal Caloric Restriction Implemented during the Preconceptional and Pregnancy Period Alters Hypothalamic and Hippocampal Endocannabinoid Levels at Birth and Induces Overweight and Increased Adiposity at Adulthood in Male Rat Offspring

**DOI:** 10.3389/fnbeh.2016.00208

**Published:** 2016-11-01

**Authors:** María Teresa Ramírez-López, Mariam Vázquez, Laura Bindila, Ermelinda Lomazzo, Clementine Hofmann, Rosarío Noemí Blanco, Francisco Alén, María Antón, Juan Decara, Rocío Arco, Daniel Ouro, Laura Orio, Juan Suárez, Beat Lutz, Raquel Gómez de Heras, Fernando Rodríguez de Fonseca

**Affiliations:** ^1^Departamento de Psicobiología, Facultad de Psicología, Universidad Complutense de MadridMadrid, Spain; ^2^Instituto de Investigación Biomédica de Málaga (IBIMA), Unidad de Gestión Clínica de Salud Mental, Hospital Regional Universitario de Málaga, Universidad de MálagaMálaga, Spain; ^3^Institute of Physiological Chemistry, University Medical Center of the Johannes Gutenberg University of MainzMainz, Germany

**Keywords:** maternal undernutrition, endocannabinoids, hypothalamus, hippocampus, rat, development, metabolism, behavior

## Abstract

Exposure to inadequate nutritional conditions in critical windows of development has been associated to disturbances on metabolism and behavior in the offspring later in life. The role of the endocannabinoid system, a known regulator of energy expenditure and adaptive behaviors, in the modulation of these processes is unknown. In the present study, we investigated the impact of exposing rat dams to diet restriction (20% less calories than standard diet) during pre-gestational and gestational periods on: (a) neonatal outcomes; (b) endocannabinoid content in hypothalamus, hippocampus and olfactory bulb at birth; (c) metabolism-related parameters; and (d) behavior in adult male offspring. We found that calorie-restricted dams tended to have a reduced litter size, although the offspring showed normal weight at birth. Pups from calorie-restricted dams also exhibited a strong decrease in the levels of anandamide (AEA), 2-arachidonoylglycerol (2-AG), arachidonic acid (AA) and palmitoylethanolamide (PEA) in the hypothalamus at birth. Additionally, pups from diet-restricted dams displayed reduced levels of AEA in the hippocampus without significant differences in the olfactory bulb. Moreover, offspring exhibited increased weight gain, body weight and adiposity in adulthood as well as increased anxiety-related responses. We propose that endocannabinoid signaling is altered by a maternal caloric restriction implemented during the preconceptional and pregnancy periods, which might lead to modifications of the hypothalamic and hippocampal circuits, potentially contributing to the long-term effects found in the adult offspring.

## Introduction

The World Health Organization has alerted about the increased prevalence of non-communicable diseases such as obesity, hypertension or diabetes, and has pointed out the importance to implement effective interventions in order to avoid the harmful consequences of these pathologies (World Health Organization, 2014). Concerning measures of prevention and treatment, it is important to take into consideration that many of these diseases might have an early origin. Indeed, several epidemiological studies have demonstrated a correlation between reduced fetal growth and the development of features of metabolic syndrome later in life (Barker and Osmond, 1988; Hales et al., 1991; Barker et al., 1993). These data together with the evidence from animal studies, led to postulate that early nutritional insults could have an impact on the health later in life, through a process known as nutritional programming (Lucas, 1991; Barker, 2007).

Decreased weight gain during pregnancy and poor maternal nutritional conditions have been associated to low weight at birth (Institute of Medicine, 2009). In Western societies, despite the fact that excessive gestational weight gain is far more common (Gould Rothberg et al., 2011), the consequences of weight gain below the recommendations should be contemplated as well. For instance, women displaying eating disorder symptoms may not be able to reach an adequate gestational weight gain according to their pre-gestational body mass index (Treasure and Russell, 1988; Micali et al., 2007; Institute of Medicine, 2009), which may lead to increased risk of preterm births or delivery of babies of smaller size (Linna et al., 2014). Additionally, in obese and normoweight women, decreased weight gain during pregnancy may reduce the risk of suffering from maternal obstetrical complications but also increase the possibility of newborns small for their gestational age (Kapadia et al., 2015). Consequently, an optimal weight gain during pregnancy seems critical to avoid adverse neonatal outcomes. Therefore, monitoring the maternal nutritional status in the early stages of the pregnancy, including the periconceptional period, and determining whether the offspring are at risk of developing metabolic disorders later in life represent a crucial point to investigate.

Inadequate nutritional conditions during both preconceptional period and early stages of embryonic development can strongly affect the programming process. Animal models of maternal undernutrition have provided further biological information on the long-term consequences of disturbing normal nutritional patterns. These studies have identified that both the pre/periconceptional and gestational malnutrition resulted in an outcome consistent with the metabolic and behavioral effects observed in humans exposed to undernutrition in critical windows of development. Thus, studies adopting animal models of suboptimal *in vitro* fertilization/culture have demonstrated robust metabolic alterations resulting in obesity and hepatic steatosis in the offspring (Fernández-Gonzalez et al., 2004; Serrano et al., 2014). Additionally, evidence from animal models have shown that either caloric restriction or weight loss during the periconceptional/gestational period could lead to epigenetic alterations (Raychaudhuri et al., 2008), developmental impairment of the hypothalamus (Sebert et al., 2011), altered leptin signaling (Bouret et al., 2004), alterations in blood pressure, adrenocortical growth or hypothalamus-pituitary adrenal axis in the offspring (Edwards and McMillen, 2002; McMillen et al., 2004; Zhang et al., 2013). These events may occur independently of the pre-pregnancy maternal body weight or whether an adequate maternal nutrition is provided in the later phases of pregnancy.

In addition to the metabolic dysregulations resulting from an inadequate programming, behavioral abnormalities in offspring after altered nutritional conditions during pregnancy have been reported. For instance, suboptimal *in vitro* culturing of embryos resulted in anxiety and learning deficits in adults (Fernández-Gonzalez et al., 2004), whereas caloric restriction during pregnancy altered feeding behavior, induced hyperphagia (Breton et al., 2009; Palou et al., 2010), modified food preferences (Lussana et al., 2008; Palou et al., 2010; Lukaszewski et al., 2011) and impaired anxiety-related behaviors in several species, including humans (Erhard et al., 2004; Nomura et al., 2007; Akitake et al., 2015).

Although as described above, many signaling systems can be affected by maternal undernutrition, to date, little is known about the contribution of the endocannabinoid system in these processes, despite the connection of this system with crucial biologic pathways, such as leptin (Di Marzo et al., 2001), and its involvement in energy metabolism, feeding behavior and emotional control (Lutz, 2009; Alén et al., 2013; Lutz et al., 2015). The endocannabinoid system plays a pivotal role in brain development (Maccarrone et al., 2014) and the disruption of endocannabinoid signaling in early stages of development has been associated to disturbances in neuronal activity, deficient cortical connections and behavior alterations (Rodríguez de Fonseca et al., 1991; Antonelli et al., 2005; Bernard et al., 2005; Moreno et al., 2005; de Salas-Quiroga et al., 2015). Moreover, the type of diet can modify the endocannabinoid content in different tissues, including the brain during development (Berger et al., 2001; Matias et al., 2003; Ramírez-López et al., 2016). This could be explained in part because the two main endocannabinoids, anandamide (AEA) and 2-arachidonoylglycerol (2-AG), derive from arachidonic acid (AA). Moreover, the arachidonic acid is a derivative of the linoleic acid, an essential dietary n-6 fatty acid (Hansen and Artmann, 2008). Therefore, an inadequate maternal diet might alter the endocannabinoid signaling and disturb the establishment of circuitries regulating metabolism, learning and emotional control, leading to metabolic and neurobehavioral abnormalities later in life (Keimpema et al., 2013; Ramírez-López et al., 2016). However, further investigation is necessary to clarify the role of the endocannabinoid system in these processes.

Based on this evidence, in the present study, we investigated the impact of a moderate maternal caloric restriction, implemented during the preconceptional and pregnancy period, on the levels of endocannabinoids and N-acylethanolamides (NAEs) in brain structures involved in metabolism and emotional responses, such as hypothalamus, hippocampus and olfactory bulb at birth. The neonatal outcomes were also assessed and the offspring were followed until adulthood to determine the impact of maternal diet on metabolism-related parameters and behavior. We propose that endocannabinoid signaling can be altered by a moderate calorie-restricted diet implemented before pregnancy and during pregnancy, and this alteration might be associated to metabolic and behavior abnormalities in the offspring later in life.

## Materials and Methods

This study was approved by the Animal Ethics Committee of the Complutense University of Madrid. Additionally, experiments were performed in compliance with Spanish regulations (RD 53/2013 178/2004) and with the European Directive 2010/63/EU on the protection of animals used for scientific purposes.

### Animals, Diets and Experimental Design

The study was carried out initially in adolescent female Wistar rats (Harlan, Barcelona, Spain). Animals were allowed to acclimate for at least 4 weeks before the diet assignation. Rats were handled and individually housed at a 12 h light-dark cycle with temperature of 22 ± 1°C. Two weeks before the diet assignation, food intake and body weight gain were monitored weekly. After the acclimatization period, animals were randomly assigned to control (*n* = 9) or caloric restriction diet (*n* = 15). At this stage, no statistical significant difference in body weight among groups was found (average weight 240.7 ± 3.4 g).

Control rats were given free access to standard chow (Standard chow SAFE A04, Panlab, Barcelona, Spain). The standard chow provided 16.1% of protein, 60% carbohydrate, 3.1% fat, 4% fiber, 0.0025% sodium and 2.9 kcal/g as energy content. In contrast, calorie-restricted dams were given a daily amount of food corresponding to 80% of the caloric intake of control dams, which was adjusted according to the body weight (20% of caloric restriction). We selected this food restriction schedule as a moderate calorie-restricted diet has been demonstrated to be sufficient to induce long-lasting alterations in offspring without affecting fertility (Terry et al., 2005; Palou et al., 2010). Water was provided *ad libitum* in both animal groups.

From the beginning of the experiment, food intake and weight were measured daily and cumulative weight gain and caloric intake were calculated weekly. Estrous cycle was evaluated every day to check cycle regularity and duration of every stage. Two weeks after the beginning of the experimental diets, females were allowed to mate with a male of the same strain. Each male rat was mated with females from both groups. The mating phase occurred in the female cage within 24 h from the proestrus. During the mating phase, food restriction was abolished and animals were allowed to eat *ad libitum* in order to avoid misinterpretation of individual food consumption or interferences in copulation (Terry et al., 2005; Klingerman et al., 2011). Twenty-four hours after proestrus, the males were returned to their cage and restricted dams carried on with their assigned diets. Then, vaginal smear was evaluated to detect the presence of vaginal plug or spermatozoa. The day these signs of successful mating were found, which confirmed pregnancy, was designated as gestational day 0 (GD 0). In the case of no pregnancy signs, the overall procedure was repeated in the following proestrus period.

**Figure 1:**
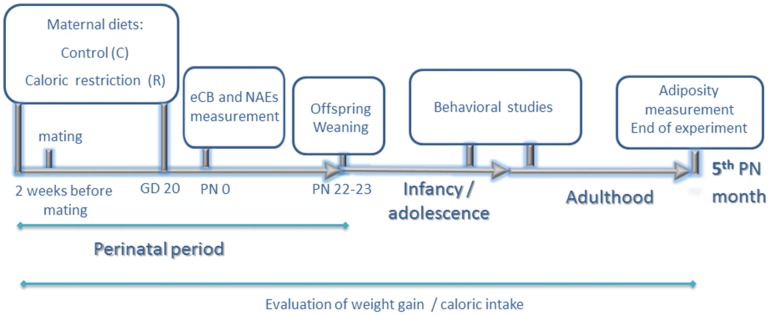
**Experimental design.** Experiments started 2 weeks before mating. Calorie-restricted rat dams received 80% of calories provided to the control group daily according to body weight (food restriction of 20%). Two weeks later, they were allowed to mate with males of the same strain. Food restriction continued up to gestational day 20 (GD 20). At birth postnatal day 0 (PN0), endocannabinoid and N-acylethanolamide (NAE) levels of male offspring were measured in hypothalamus, hippocampus and olfactory bulb. During lactation, rat dams from both groups continued on standard chow diet *ad libitum*. At PN 22–23 day, offspring were weaned on standard chow diet and rat dams were sacrificed. Behavioral studies (elevated plus maze, open field and chocolate preference test) were performed at adolescence (8th PN weeks). Chocolate preference was also reevaluated at adulthood (12–13th PN weeks). Adiposity was measured at 5th postnatal month. Caloric intake and weight gain were evaluated in rat dams and offspring during the entire duration of the experimental procedure.

During the gestational period, food intake and weight were measured daily and female rats were maintained on the calorie-restricted diet until the GD 20, 2 days previous to birth (GD 20), when restricted diet ended (Figure 1). The birth day was defined as postnatal day 0 (PN0). Within 14 h after birth, pups were weighed and sexed. Litter size was adjusted to eight pups, consisting of four males and four females. The remaining pups were quickly sacrificed by decapitation and brains were collected for further endocannabinoid analysis.

Throughout the lactation period, calorie-restricted and control rat dams were provided with standard chow *ad libitum*. Food intake and dams’/pups’ weight were measured 3 days per week during this period. At PN 22–23, offspring from both types of perinatal diet were weaned onto standard chow diet (*n* = 15 and *n* = 23, for control and caloric restriction group, respectively). Rats belonging to the same litter from each perinatal diet group were housed together (2–3 rats/cage). Dams were sacrificed. During the post-weaning period, weight and food intake were measured weekly. Behavioral studies were performed in adolescence and adulthood (Figure 1). At the 5th postnatal month, three quarters of the male offspring were sacrificed.

All experiments in offspring were performed in males. The term “perinatal” was used to refer to pregestational, gestational and lactation periods.

### Evaluation of Parameters Related to Fertility

The evaluation of estrous cycle started 2 weeks before mating and it was performed daily until post-mating day. Every morning, in the 2 h following the beginning of the dark phase, each female was moved to the experimental room. A pipette filled with 0.2 ml of clean water was inserted gently in the vagina. Then, vaginal fluid was collected by aspiration and one drop of it was placed on a glass slide and observed under a light microscope. The stages of estrous cycle identified were proestrus, estrus, metestrus and diestrus (Marcondes et al., 2002). Prolonged diestrus was considered when diestrus lasted for three consecutive days or more.

### Caloric Intake and Adiposity

Food intake was determined by subtracting the amount of each food type left in the cage from the total amount of food provided. In weaned offspring, the individual food intake was determined by dividing the total food intake from each cage by the number of pups per cage. Comparisons among groups were carried out by calculating cumulative caloric intake relative to body weight (Kcal/Kg) as well as weight gain in each period of the study. Furthermore, the percentage of increase in body weight and caloric intake in rat dams was calculated at the end of the caloric restriction diet and specifically, on GD 20 and 21 and during the lactation period, from PN 1–22.

Adiposity was estimated by calculating the percentage of abdominal fat weight over total body weight at the time animals were sacrificed. Rats were weighed immediately before death and sacrificed by decapitation after administration of Equitesin^®^ (3 mg/kg). Then, perirenal and perigonadal fat deposits were dissected and weighed. The sum of both types of fat was used to determine the percentage of abdominal fat.

### Endocannabinoid Measurements

At PN0, male offspring chosen to be sacrificed from the two experimental groups were decapitated during the 2nd/3rd h of the dark phase and brains were quickly removed and frozen at −80°C until brain region isolation. To avoid the possibility of variable outcomes among litters, brains from at least three litters per group were used to carry out endocannabinoid measurements (control pups *n* = 7–5–3 and pups from restricted dams *n* = 16–11–9, for hypothalamus, hippocampus and olfactory bulb, respectively). For the isolation of the brain regions selected, brains were thawed in cold Tris-HCl buffer (50 mM, pH = 7.40) and the entire hypothalamus, right hippocampus and right olfactory bulb were quickly isolated and immediately frozen at −80°C until lipid extraction. The overall isolation procedure was carried out in less than 7 min for all animals to avoid *ex vivo* production/degradation of endocannabinoids.

For lipid extraction, pre-cooled steel balls of 5 mm were added to pre-cooled tubes containing the tissue. A solution of deuterated endocannabinoids (AEA-d4, 2-AG-d5, AA-d8, MAEA, oleoylethanolamide (OEA)-d2, palmitoylethanolamide (PEA)-d4 and 1-AG-d5, Cayman Chemicals, Ann Arbor, MI, USA) in acetonitrile was added to the tissue along with 300 μL of ice-cold 0.1 M formic acid and 300 μl of ethylacetate/hexane (9:1, v/v). Then, the samples were homogenized with a TissueLyser II (Qiagen, Hilden, Germany) for 60 s at 30 Hz. Subsequently, the samples were centrifuged for 10 min at 5000 g and 4°C. The organic phase was removed and evaporated under a gentle stream of nitrogen at 37°C. The aqueous phase was further used for protein content determination. The lipid extract was re-dissolved in 50 μL acetonitrile/water (1:1, v/v) and quantitative analysis of the endocannabinoid levels was carried out by liquid chromatography-multiple reaction monitoring (LC-MRM). The concentrations of internal standards, as well as the calibration curves, were set and tailored using test hypothalamic hippocampal and olfactory bulb tissues. LC/MRM conditions for quantitative analysis of the endocannabinoids were set as endocannabinoid levels were normalized to the corresponding protein content of the tissues as previously reported (Wenzel et al., 2013; Bindila and Lutz, 2016).

For protein quantification, the BCA method (bicinchoninic acid assay) was used and the measurements performed by using a FLUOstar Galaxy (BMG Lab technologies).

## Behavioral Studies

### Elevated Plus Maze

Anxiety-related behaviors were evaluated in handled animals with the elevated plus maze, at the 7–8th PN weeks (adolescence period). The elevated plus-maze (Panlab, Barcelona, Spain) consisted of a cross-shaped platform made of black and gray plastic. The platform was elevated 65 cm from the floor and had two opposing open arms (50 cm × 10 cm) and two closed arms of the same size. A central area of 10 cm connected all arms. The closed arms were fenced by 50-cm high opaque walls. The light intensity was adjusted at 150 lux in the open arms and 80 lux in the closed arms. The test was performed after 5 h from the beginning of the dark phase. When the test started, each rat was placed in the central area facing one of the open arms and opposite to the experimenter position. Then, the animal was allowed to explore the maze freely for 5 min. After this time, the rat was moved back to the home cage and the maze was cleaned. The number of open-arm and closed-arm entries and the percentage of time spent on open and closed arms were determined by a computer-controlled system recording the interruptions of infrared photo beams located along each arm. Data were analyzed by using the MAZE soft software (Panlab, Barcelona, Spain). Animals that fell off the maze during the test were excluded from the analysis.

### Open Field

Locomotor activity and anxiety-related behavior were evaluated with the open field test in the 2 days following the elevated plus maze test. The open field consisted of a square arena (80 cm × 80 cm and 40 cm high) virtually divided into a peripheral zone and a central zone (40 cm × 40 cm). It was made of plywood and was located in an experimental room illuminated with low light intensity (30 lux). The test was performed 5 h after the beginning of the dark phase. Each rat was positioned in the center of the open field and was allowed to explore freely for 5 min. After this time, the rat was moved back to the home cage and the arena was cleaned. A video camera installed above the arena was connected to a monitor and a video tracking motion analysis system (Smart, Panlab, Harvard Apparatus, Spain), which measured the total distance traveled (cm) and mean speed (cm/s). The program calculated the percentage of time spent in the central area as well as the number of entries to the center zone, as an index of anxiety-like behavior.

### Chocolate Preference Test

The chocolate preference test was performed at adolescence (8–9th PN weeks) and adulthood (12–13th PN weeks). At the beginning of the test, animals were single-housed in new cages provided with both types of food (standard chow and the mixture of chocolates) and water *ad libitum*. Food intake for both types of food and animal weight was determined 24 h after the beginning of the test. Chocolate preference was calculated as the percentage of chocolate eaten over total food provided, a measure that was independent of the body weight of the animal and that only reflects food preference.

### Statistical Analysis

All data are expressed as mean ± SEM. Statistical analysis of results was performed by using the GraphPad Prism version 5.0 program (GraphPad Software Inc., San Diego, CA, USA) and SPSS 15.0 for windows (SPSS Inc., Chicago, IL, USA). Weight gain over the time and caloric intake were analyzed by one-way repeated measures analysis of variance (ANOVA). Multiple comparisons were assessed by Bonferroni *post hoc* test. To assess the differences among groups on fertility-related parameters the chi-squared test was adopted. Results from the chocolate preference test were analyzed by two-way ANOVA with group (control vs. free-choice animals) and age period used as variables. Further analyses were performed by using the Student *t-*test, when data passed normality requirements (D’Agostino-Pearson test) or U Mann Whitney test. A *p*-value below 0.05 was considered statistically significant.

## Results

### Impact of Caloric Restriction on Rat Dams During the Perinatal Period

#### Impact of Caloric Restriction on Fertility Parameters

The evaluation of estrous cycle was performed daily during the 2 weeks before mating in order to detect alterations in regularity and duration of the different stages of the estrous cycle.

After the beginning of the experiment, 6 over 15 calorie-restricted dams suffered from prolonged diestrus or irregular cycle, whereas only 1 over 9 control rat dams did. However, chi-squared test did not show significant differences between groups ($x_{\text{df}1}^{2}$ = 2.272, *p* > 0.05; data not shown). Regarding the number of days required to become pregnant, dams assigned to restricted diet had higher tendency to spend 2 days or more with a male in proestrus (chi-squared test: $x_{\text{df}1}^{2}$ = 3.646, *p* = 0.056; data not shown). However, successful mating was finally achieved in both experimental groups.

These results indicate that a moderate preconceptional caloric restriction do not affect fertility, despite the subtle changes observed during the estrous cycle and mating phase.

#### Effect of Caloric Restriction on Maternal Weight Gain

**Figure 2:**
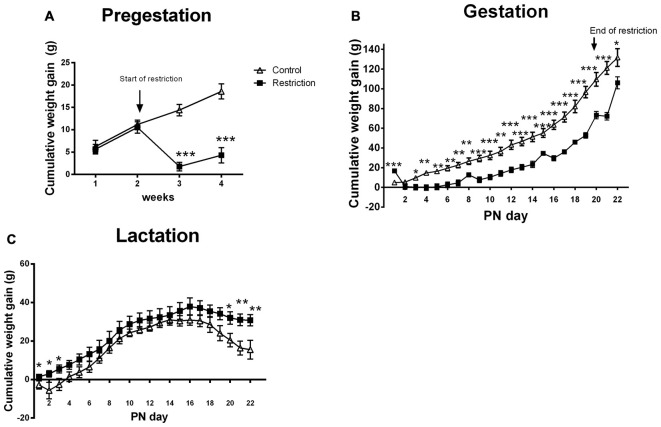
**Maternal weight gain during pregestation, gestation and lactation period.** Cumulative weight gain (g) of control (open triangles) and restricted dams (solid squares) during pregestation **(A)** gestation **(B)** and lactation **(C)**. Values are expressed as means ± SEM. *p < 0.05, **p < 0.01, ***p < 0.001.

Repeated measures ANOVA revealed that dams from caloric restriction group gained less weight than controls before pregnancy (*F*_(1,23)_ = 19.538, *p* < 0.001). The differences started to be significant after the first week from the beginning of the restriction diet (*F*_(1,23)_ = 65.361, *p* < 0.001; Figure 2A). At mating, calorie-restricted dams tended to weigh less as compared to control dams (mean weight control vs. restriction: 250.1 ± 8.921 vs. 235.4 ± 2.882; Student’s *t*-test control vs. restriction group: *t* = 1.886, *p* = 0.07, data not shown).

During the entire gestational period, the cumulative weight gain was decreased in the caloric restriction group (repeated measures ANOVA: *F*_(1,20)_ = 23.902, *p* < 0.001). However, Bonferroni multiple comparisons showed that restricted dams gained more weight at GD 0 (*F*_(1,20)_ = 23.9, *p* < 0.001), probably because they were allowed to eat *ad libitum* during the mating phase. Moreover, the differences in weight gain between groups were mitigated at GD 21, when restricted diet ended and calorie-restricted pregnant dams were allowed to eat *ad libitum* (*F*_(1,20)_ = 5.526, *p* < 0.05; Figure 2B). Accordingly, the percentage of increase in body weight between GD 20 and 21 was higher for restricted dams as compared to controls (U Mann Whitney test, *U* = 2.000, *p* < 0.001; Data not shown). Despite the change in body weight at the end of diet restriction, at PN0 calorie-restricted dams still tended to be leaner than controls (mean weight and SEMs control vs. restriction: 291 ± 12.06 (*n* = 9) vs. 273 ± 2.95 (*n* = 15); Student’s *t*-test: *t* = 1.820, *p* = 0.08; data not shown).

Regarding the cumulative weight gain during the entire lactation period, no significant differences between groups were found (ANOVA of repeated measures, *F*_(1,22)_ = 2.389, *p* = 0.136). However, Bonferroni multiple comparisons showed that calorie-restricted dams gained more weight at the beginning and at the end of lactation as compared to controls (*F*_(1,22)_ = 4.811, *p* < 0.05; *F*_(1,22)_ = 4.850, *p* < 0.05; *F*_(1,22)_ = 5.061, *p* < 0.05; *F*_(1,22)_ = 5.258, *p* < 0.05; *F*_(1,22)_ = 9.428, *p* < 0.01 and *F*_(1,22)_ = 8.301, *p* < 0.01, at PN day 1, PN day 2, PN day 3, PN day 20, PN day 21 and PN day 22, respectively; Figure 2C). Consequently, the percentage of increase in body weight between PN day 1 and 22 was higher for restricted dams than controls (5.578% ± 1.83% vs. 11.24 ± 1.076%; Student’s *t*-test, *t* = 2.854, *p* < 0.01; data not shown).

#### Effect of Caloric Restriction on Maternal Caloric Intake

**Figure 3:**
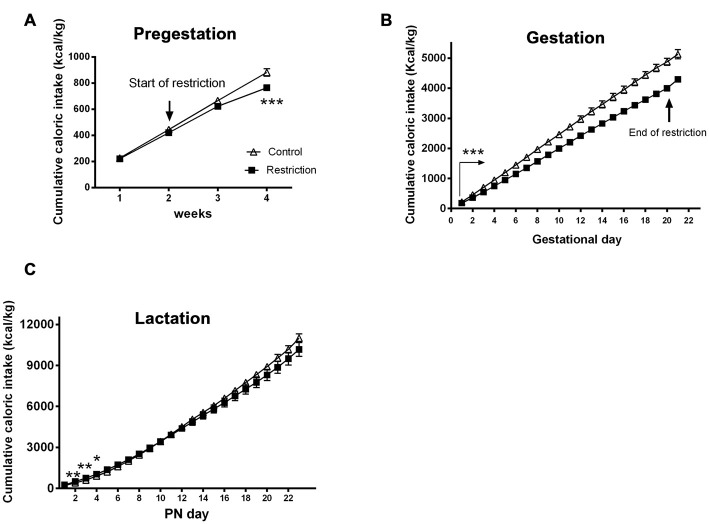
**Maternal caloric intake during pregestation, gestation and lactation period.** Cumulative caloric intake (g) of control (open triangles) and restricted dams (solid squares) during pregestation **(A)** gestation **(B)** and lactation **(C)**. Values are expressed as means ± SEM. *p < 0.05, **p < 0.01, ***p < 0.001. The arrow in **(B)** with statistical points ***denotes the interval of time with significant differences between groups (*p* < 0.001).

Repeated measures ANOVA demonstrated that cumulative caloric intake in rat dams from diet restriction group was significantly lower than controls before pregnancy (*F*_(1,23)_ = 8.798, *p* < 0.01; Figure 3A). These differences were particularly evident after 2 weeks from the beginning of the restricted diet (*F*_(1,23)_ = 20.228, *p* < 0.001; Figure 3A), as revealed by Bonferroni multiple comparisons.

During the overall gestational period, calorie-restricted dams continued consuming less calories per kg (repeated measures ANOVA *F*_(1,20)_ = 62.491, *p* < 0.001; Figure 3B). As restricted mothers were allowed to eat *ad libitum* at day 20, they presented increased caloric intake compared to controls between GD 20 and 21 (Student’s *t*-test: *t* = 4.550, *p* < 0.001; data not shown).

Additionally, repeated measures ANOVA indicated no significant differences between groups in cumulative caloric intake (g/kg) during the entire lactation period (*F*_(1,22)_ = 0.330, *p* > 0.05). However, the days immediately after birth, restricted mothers tended to be hyperphagic (*F*_(1,22)_ = 3.045, *p* = 0.09; *F*_(1,22)_ = 9.523, *p* < 0.01; *F*_(1,22)_ = 11.273, *p* < 0.01, *F*_(1,22)_ = 4.896, *p* < 0.05 and *F*_(1,22)_ = 3.365, *p* = 0.08, at PN day 1, PN day 2, PN day 3, PN day 4 and PN day 5, respectively; Figure 3C).

### Effect of Maternal Caloric Restriction on Birth Outcomes

Control pups were born between GD 21 or 22, whereas all pups from calorie-restricted dams were born at GD 22 (data not shown). All pups were born during the light phase. At birth, Student’s *t*-test analysis revealed that offspring from preconceptional/pregnancy calorie-restricted dams did not differ in weight from control pups, either when analysis was done in both sexes together or in male and female separately (data not shown). However, litter size tended to be lower in the diet restriction group (11.67 ± 0.81 vs. 8.73 ± 1.01; Student’s *t*-test, *t* = 2.005, *p* = 0.05). Indeed, a significant decreased little size was found in female (6.111 ± 0.67 vs. 4.067 ± 0.49, Student’s *t*-test, *t* = 2.484, *p* < 0.05) but not in male pups.

### Effect of Maternal Caloric Restriction on Brain Endocannabinoid and N-acylethanolamide Levels in Newborn Male Offspring

#### Hypothalamic Endocannabinoid and N-acylethanolamide Levels in Male Offspring at Birth

**Figure 4:**
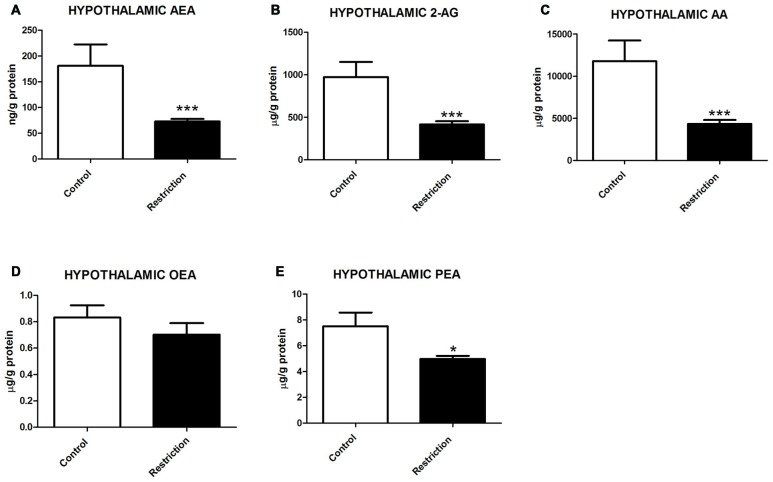
**Hypothalamic endocannabinoid and NAE levels in male offspring at birth. (A)** Anandamide (AEA), **(B)** arachidonoylglycerol (2-AG), **(C)** Arachidonic acid (AA), **(D)** oleoylethanolamide (OEA), and **(E)** palmitoylethanolamide (PEA) in the hypothalamus of male offspring from control dams (open bars) and calorie-restricted dams (solid bars) at birth.*p < 0.05, ***p < 0.001.

U Mann Whitney test showed significant differences between groups in hypothalamic endocannabinoids and/or NAEs at birth. Specifically, male pups from calorie-restricted dams presented significantly lower levels of AEA (*U* = 2.000, *p* < 0.001), 2-AG (*U* = 3.000, *p* < 0.001) and AA (*U* = 4.000, *p* < 0.001; Figures 4A–C, respectively). Concerning NAE levels, no significant differences between groups was found in OEA levels (*U* = 35.50, *p* > 0.05; Figure 4D), although PEA levels were significantly decreased in offspring from calorie-restricted dams as compared to controls (*U* = 17.00, *p* < 0.05; Figure 4E).

These results indicate that a prolonged caloric restriction during the preconceptional/pregnancy period induces a strong decrease in the levels of the predominant endocannabinoids (AEA and 2-AG), their precursor (AA) as well as in PEA levels in the hypothalamus of offspring at PN0.

#### Hippocampal Endocannabinoid and N-acylethanolamide Levels in Male Offspring at Birth

**Figure 5:**
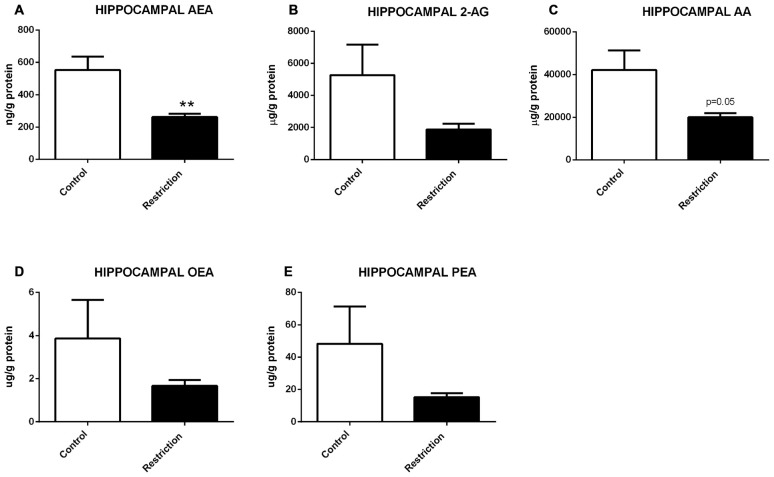
**Hippocampal endocannabinoid and NAE levels in male offspring at birth. (A)** Anandamide (AEA), **(B)** arachidonoylglycerol (2-AG), **(C)** Arachidonic acid (AA), **(D)** oleoylethanolamide (OEA), and **(E)** palmitoylethanolamide (PEA) in the hippocampus of male offspring from control dams (open bars) and calorie-restricted dams (solid bars) at birth. **p < 0.01.

U Mann Whitney test showed a general propensity to reduced endocannabinoid levels in the hippocampus of offspring from calorie-restricted dams as compared to controls. Specifically, male pups from diet-restricted dams presented significantly decreased levels of AEA (*U* = 9.000, *p* < 0.01; Figure 5A), a strong tendency to decreased levels of AA (*U* = 16.00, *p* = 0.05; Figure 5C) and a subtle but not significant reduction of PEA (*U* = 19.00, *p* = 0.09; Figure 5E). No statistical differences between groups were found either in 2-AG or OEA levels (Figures 5B,D, respectively).

Therefore, a prolonged caloric restriction during the preconceptional/pregnancy period predominantly induces decreased levels of AEA in the hippocampus of the offspring at birth.

#### Endocannabinoid and N-acylethanolamide Levels in the Olfactory Bulb of Male Offspring at Birth

**Figure 6:**
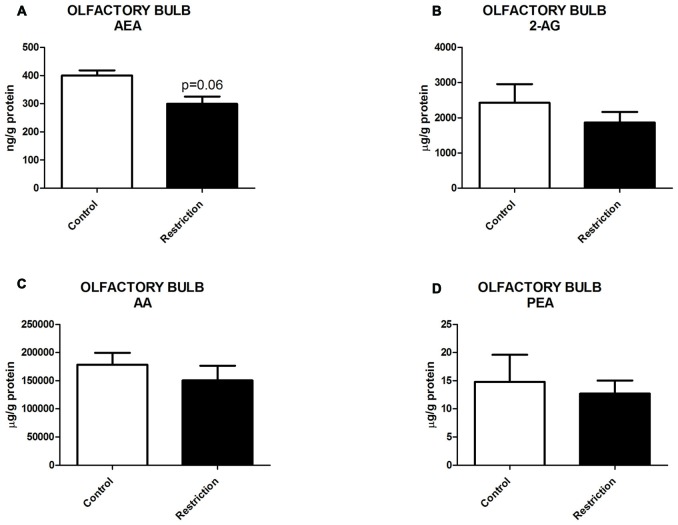
**Endocannabinoid and NAE levels in the olfactory bulb of male offspring at birth. (A)** Anandamide (AEA), **(B)** arachidonoylglycerol (2-AG), **(C)** Arachidonic acid (AA) and **(D)** palmitoylethanolamide (PEA) in the olfactory bulb of male offspring from control dams (open bars) and calorie-restricted dams (solid bars) at birth. Values are expressed as mean ± SEM.

U Mann Whitney test showed a slight tendency to decreased levels of AEA in the olfactory bulb of offspring from calorie-restricted dams (Figure 6A). No significant differences between groups were found in 2-AG, AA and PEA levels (Figures 6B–D). The OEA values in olfactory bulb could not be reliably quantified due to interference of a peak corresponding to an isobaric structure of OEA (data not shown).

### Effect of Maternal Caloric Restriction on Offspring Metabolic-Related Parameters After Birth

#### Effect of Maternal Diet on Male Offspring Weight Gain During Lactation Period

**Figure 7:**
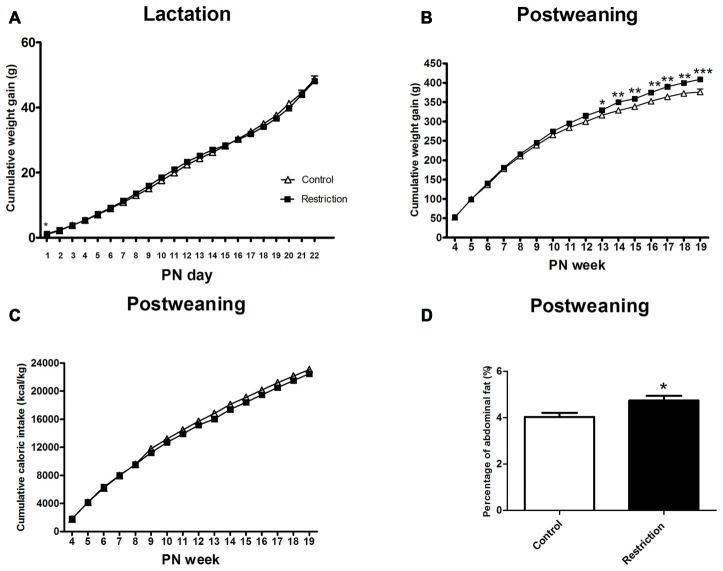
**Effect of preconceptional/pregnancy restricted diet on metabolic-related parameters in male rat offspring after birth.** Cumulative weight gain (g) of control group offspring (open triangles) and caloric restricted group offspring (solid squares) during lactation **(A)** and postweaning **(B)** period. Cumulative caloric intake (Kcal/kg) of control offspring (open triangles) and calorie-restricted offspring (solid squares) during postweaning period **(C)**. Offspring adiposity, calculated as the percentage of the abdominal fat (the sum of perirenal and perigonadal fat) at 5th PN month, is shown in **(D)**: control offspring (open bars) vs. caloric restricted offspring (solid bars). Values are expressed as mean ± SEM. *p < 0.05, **p < 0.01, ***p < 0.001.

Repeated measures ANOVA indicated no differences between groups in weight gain during the lactation period (*F*_(1,81)_ = 0.021, *p* > 0.05). However, at PN day 1 pups from calorie-restricted dams gained more weight than controls (*F*_(1,81)_ = 4.024 *p* < 0.05; Figure 7A).

#### Post-Weaning Weight Gain, Caloric Intake and Adiposity

After weaning, repeated measures ANOVA revealed a significant effect of the perinatal diet on male weight gain (*F*_(1,31)_ = 6.132, *p* < 0.05) and a statistically significant interaction between time and perinatal diet (*F*_(15,17)_ = 3.212, *p* < 0.05; Figure 7B). Indeed, Bonferroni multiple comparisons showed that offspring from preconceptional/pregnancy diet restricted mothers started to gain more weight at the beginning of adulthood and specifically at the 13th PN week. (*F*_(1,31)_ = 4.693, *p* < 0.05; *F*_(1,31)_ = 8.547, *p* < 0.01; *F*_(1,31)_ = 9.350,*p* < 0.01; *F*_(1,31)_ = 9.806, *p* < 0.01, *F*_(1,31)_ = 12.876, *p* < 0.05; *F*_(1,31)_ = 13.227, *p* < 0.01 and *F*_(1,31)_ = 17.014, *p* < 0.001, at 13th PN week, 14th PN week, 15th PN week, 16th PN week, 17th PN week, 18th PN week and 19th PN week, respectively; Figure 7B).

Regarding the variable absolute body weight, repeated measures ANOVA did not show a significant main effect of the perinatal diet on male offspring body weight (*F*_(1,36)_ = 1.185, *p* = 0.284), when considering the entire period as statistical variable. However, the interaction between time and body weight was statistically significant (*F*_(1,36)_ = 2.684, *p* < 0.05). Thus, Bonferroni multiple comparisons revealed that the absolute body weight of offspring from calorie-restricted mothers started to be significantly higher at the 15th PN week (*F*_(1,36)_ = 5.024, *p* < 0.05; *F*_(1,36)_ = 2.539,*p* = 0.120; *F*_(1,36)_ = 4.451, *p* < 0.05; *F*_(1,36)_ = 4.642, *p* < 0.05; *F*_(1,36)_ = 4.642, *p* < 0.05 and *F*_(1.36)_ = 6.934, *p* < 0.05, at 15th PN week, 16th PN week, 17th PN week, 18th PN week and 19th PN week, respectively; data not shown).

Concerning caloric intake, repeated measures ANOVA indicated no statistical differences between groups in this variable, despite the fact that offspring from calorie-restricted dams started to gain more weight in adulthood (*F*_(1,36)_ = 1.865, *p* > 0.05; Figure 7C).

Regarding adiposity, male offspring from preconceptional-gestational calorie-restricted dams presented a statistically significant increase in abdominal fat as compared to control offspring at the 5th PN month (Student’s *t*-test, *t* = 2.069, *p* < 0.05; Figure 7D). These data are consistent with the increased body weight exhibited by these animals.

Taking together, these results show that male offspring from preconceptional-gestational calorie-restricted dams presented higher body weight and weight gain and increased adiposity than controls in adulthood, despite the fact that caloric intake was not changed in these animals.

### Effect of Maternal Diet on Male Offspring Behavior in Postweaning Period

#### Anxiety-Like Behavior: Elevated Plus Maze Test and Open Field Test

**Figure 8:**
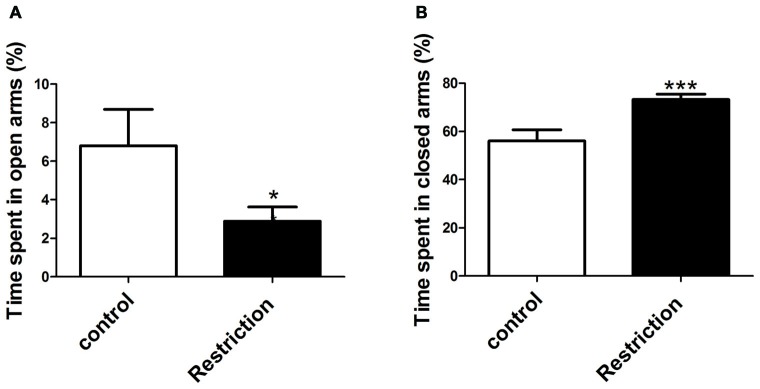
**Anxiety-related behaviors in male rat offspring.** Anxiety-related responses were evaluated through the elevated plus maze test. The percentage of time spent in open and closed arms of the elevated plus maze test is indicated in (**A,B**) respectively, and was calculated over the total test time (300 s). Values are expressed as mean ± SEM. *p < 0.05 and ***p < 0.001.

Student’s *t*-test and U Mann Whitney test analysis revealed that offspring from calorie-restricted dams showed higher propensity to display anxiety-like behaviors as compared to controls. Indeed, offspring from preconceptional calorie-restricted dams spent significantly less time in the open arms (*t* = 2.213, *p* < 0.05; Figure 8A) and longer time in the closed arms of elevated plus maze (*t* = 3.727, *p* < 0.001; Figure 8B). However, no differences were found either in the percentage of number of entries in the open arms or in the percentage of entries in the closed arms of the elevated plus maze (data not shown). Regarding the open field test, U Mann Whitney and Student’s *t*-test analysis showed that offspring from restricted dams tended to spend less time in the central area of the arena (*U* = 63.00, *p* = 0.08). However, no significant differences between groups were found in the number of entries in the central zone of the arena (*t* = 0.06, *p* > 0.05; data not shown).

#### Locomotor Activity: Open Field Test

Student’s *t*-test analysis showed no significant differences between groups in the total distance traveled in the open field test. In addition, the mean speed was not statistically different between groups (data not shown). These results suggest that alterations in the perinatal diet do not affect locomotor activity in the offspring.

#### Chocolate Preference

Two way ANOVA test performed with the chocolate preference test carried out in adolescence and adulthood, showed a statistically significant effect for the factor age period (adolescence vs. adulthood; *F*_(1,68)_ = 32.220, *p* < 0.001). Thus, the preference for chocolate was increased over time in all groups. In contrast, no significant differences in the main effect of perinatal diet and no significant interaction between factors (perinatal diet × age time) were found. However, the increment for chocolate preference by age period was slightly different between groups as revealed by Bonferroni multiple comparisons. Indeed, although offspring from control dams showed increased chocolate preference in adulthood (*F*_(1,68)_ = 11.613, *p* < 0.01), offspring from calorie-restricted dams displayed a more pronounced increase for chocolate preference by age period (*F*_(1.68)_ = 23.353, *p* < 0.001; Data not shown).

## Discussion

In the present study, we have demonstrated that a maternal caloric restriction implemented during the preconceptional/pregnancy period until 2 days prior birth, alters the weight gain during pregnancy and affects the litter size without changing offspring weight at birth. Importantly, this is the first study to demonstrate that this moderate caloric restriction decreases endocannabinoid and/or NAE levels in hypothalamus and hippocampus in male offspring at birth, and that these alterations are associated to overweight, increased adiposity and anxiety-related responses in adulthood. These findings suggest the need for considering the endogenous cannabinoid system as a relevant signaling system involved in metabolic/behavioral programming.

It is important to note that these findings were obtained using a model of moderate caloric restriction that does not affect fertility, but is sufficient to produce changes in the litter size and the body weight gain of dams (Terry et al., 2005; Palou et al., 2010). This is relevant because more severe diet restriction may have led to increased pre and postimplantational fetus loss. In any case, the weight loss observed along preconceptional diet restriction might have produced an impact in the uterine glands that provide nutrition during the conceptional period and the first stages of pregnancy nutrients, a process known as histiotrophic nutrition (Burton et al., 2002), which might be sufficient to generate the effects observed. In addition, suboptimal nutrient conditions might have affected the embryos, through a process similar to that described in suboptimal *in vitro* culture in rodents, as we have recently shown (Fernández-Gonzalez et al., 2004; Serrano et al., 2014). Intriguingly, despite the decreased maternal weight gain during pregnancy, pups from calorie-restricted dams were not underweight at birth. These findings are consistent with other studies showing that the weight at birth could be unaffected when caloric restriction is implemented in the early stages of pregnancy (Sebert et al., 2009; Palou et al., 2010; Poore et al., 2010). The absence of differences in pups’ body weight may be associated to the significant increase in body weight and caloric intake of diet-restricted rat dams when the food restriction ended, which may have determined an accelerated weight gain in the fetus, as reported previously (Baik et al., 2014). This hypothesis is supported by the fact that offspring were born 2 days after switching the calorie-restricted dams to *ad libitum* feeding, suggesting that birth occurred only when fetuses reached the appropriate birth weight.

### A Moderate Maternal Caloric Restriction During the Preconceptional/Pregnancy Period Alters Hypothalamic and Hippocampal Endocannabinoid and NAE Levels in Male Offspring at Birth

The endocannabinoid system is a relevant homeostatic network of signals controlling energy expenditure and metabolism (Matias and Di Marzo, 2007; Bermudez-Silva et al., 2010). However, its role in developmental programming has not been studied in depth. To our knowledge, this is the first study investigating the levels of endocannabinoids and NAEs at birth after maternal exposure to restricted feeding during the preconceptional/pregnancy period. Interestingly, we found that offspring from calorie-restricted mothers presented in general reduced levels of endocannabinoids and their precursor, AA, in hypothalamus and hippocampus and a similar tendency in the olfactory bulb, as compared to controls. Reduced AA levels may be explained in part because AA (20:4n-6) derives from linoleic acid (18:2n-6), which is an essential fatty acid obtained from dietetic sources. Therefore, decreased availability of nutrients after a prolonged calorie-restricted maternal diet might have affected the levels of the precursor (AA) of the two predominant endocannabinoids (AEA and 2-AG). Conversely, reduced eCB levels, which are AA precursors as well, may also have determined in part the reduced levels of AA.

In the hypothalamus, we detected a strong reduction in the level of the main endocannabinoids and AA. These results are in agreement with Matias et al. (2003), showing decreased levels of AEA in weaning offspring from dams previously exposed to a maternal caloric restriction during pregnancy and/or lactation. However, in our experiments we detected more robust alterations in endocannabinoid and NAE levels likely because preconceptional diet restriction may induce more exacerbating effects in the offspring, as reported in both cohort studies (Roseboom et al., 2006) and animal models of undernutrition (Edwards and McMillen, 2002; McMillen et al., 2004; Zhang et al., 2013), despite the lack of change in weight at birth. Consistently, we found reduced endocannabinoid levels in normoweight pups. However, the lack of association between endocannabinoid levels and pup weight is in disagreement with Matias et al. (2003), showing decreased endocannabinoid levels in underweight pups. However, differently from Matias et al. (2003), we measured the endocannabinoids at birth. Calorie-restricted rat dams tended to weigh less than controls at birth and their endocannabinoid levels might not have been restored to normal. Thus, the endocannabinoid levels in new born rats might reflect the maternal status, due to the fact that long fatty acids can be transferred through the placenta and the fetus are not fully able to modify fatty acid structures (Keimpema et al., 2013). Future research will address these possibilities.

Although the endocannabinoid system has received little attention in the context of nutritional programming, proteins interacting with endocannabinoids, such as leptin and brain-derived neurotrophic factor (BDNF) have been shown to be altered after maternal exposure to undernutrition (Di Marzo et al., 2001; Keimpema et al., 2014). For instance, after maternal undernutrition, alterations in the surge of leptin and BDNF levels during specific developmental stages have been associated to disruption in the development of the hypothalamic circuit and metabolic disorders later in life (Bouret et al., 2004; Yura et al., 2005; Coupé et al., 2009). These data lead to hypothesize that the endocannabinoid system might be involved in the regulation of these processes as well. Indeed, during the prenatal and postnatal periods endocannabinoids levels reach a peak in concentration, which correlates with neurodevelopment processes occurring in these specific age stages (Berrendero et al., 1999). Moreover, alterations of endocannabinoid signaling by prenatal administration either of cannabinoid receptor agonists, such as THC (Δ^9^-Tetrahydrocanabinol), or antagonists, is associated to impairment in neuronal activity, cortical connections and emotional behavior (Rodríguez de Fonseca et al., 1991; Antonelli et al., 2005; Bernard et al., 2005; Moreno et al., 2005; de Salas-Quiroga et al., 2015). Importantly, our data suggest that prolonged prepregnancy-gestational undernutrition could have altered the normal fluctuations of endocannabinoid levels and thus, the establishment of functional circuitries involved in metabolism, ultimately leading to metabolic abnormalities later in life, as proposed by Keimpema et al. (2013). Supporting this hypothesis, we have recently analyzed the expression of cannabinoid CB1 and CB2 receptors mRNA in the hypothalamus of adult offspring born from mothers exposed to pre- and gestational moderate caloric restriction and we have found a clear upregulation in the expression of both receptors, indicating long-term alterations in the offspring as result of maternal undernutrition (Ramírez-López submitted).

Interestingly, although the levels of non-cannabinoid NAEs seem to be less influenced by diet (Hansen and Diep, 2009), we have found decreased levels of PEA in hypothalamus. PEA has been shown to exert anti-inflammatory (Hoareau and Roche, 2010) and antiobesity effects (Mattace Raso et al., 2014). Taken into account this evidence, PEA level deregulation in early life may predispose to diseases associated to inflammation, such as obesity and metabolic syndrome (Rana et al., 2007). Further research should be pursued to corroborate these possibilities.

We also found reductions in AEA levels and a strong tendency to decreased AA levels in hippocampus. It has been shown that circuits connecting hippocampus and hypothalamus are involved in the control of the hedonic aspects of eating (Petrovich, 2013) and that hippocampal endocannabinoid levels can be altered according to different types of diet (Rivera et al., 2012). For instance, decreased levels of hippocampal endocannabinioids have been reported after exposing young animals to prolonged starvation (Hanus et al., 2003). Moreover, we have recently reported decreased levels of hippocampal endocannabinoids after maternal exposure to a high-fat and low protein-containing diet (Ramírez-López et al., 2016). Taking into account that the endocannabinoids play an important role in the regulation of emotional processes and memory formation in this specific brain region (Lutz et al., 2015), these findings raise the question of whether decreased hippocampal endocannabinoid levels in critical windows of development may lead to altered emotional responses later in life (Ramírez-López et al., 2016).

Finally, in the olfactory bulb, another brain structure involved in the regulation of metabolism and feeding behavior, we only found a tendency towards decreased AEA levels, similarly to that found in the hippocampus and hypothalamus. Although these modifications were subtle, they still could have led to alterations in metabolism and feeding behavior later in life, considering the role of endocannabinoid signaling in odor perception and food intake (Soria-Gómez et al., 2014). Future studies are necessary to confirm these speculations.

### A Moderate Maternal Caloric Restriction During the Preconceptional/Pregnancy Period Influences Metabolic-Related Parameters in Offspring

Although offspring were weaned on standard chow diet, male offspring form calorie-restricted dams started to gain more weight as compared to controls at the beginning of adulthood and exhibited overweight starting from the 15th PN week. Increased weight was accompanied by augmented abdominal adiposity, although caloric intake was not changed in these animals. These long-lasting effects suggest that altered endocannabinoid levels, and presumably signaling, might be associated with inadequate wiring, which could directly trigger a pathological state. This deregulation may also induce subtle alterations in neuronal connectivity of brain structures involved in metabolism, which would eventually increase the vulnerability to diseases later on in life, as proposed by the direct/double hit hypothesis (Keimpema et al., 2013).

Our findings are consistent with other studies showing similar alterations in offspring exposed to a maternal caloric restriction and weaned on a normocaloric diet (Desai et al., 2005; Breton et al., 2009; Suzuki et al., 2010; García et al., 2011; Lukaszewski et al., 2011). Moreover, these effects have been shown either in offspring that were underweight (Desai et al., 2005) or normoweight at birth (Palou et al., 2010). Interestingly, adult obesity in previously undernourished fetus has been associated to early catch-up growth (Eriksson et al., 1999; Desai et al., 2005). Although we do not show direct evidence of early catch-up growth in calorie-restricted offspring, the fact that they were born normoweight and displayed increased weight gain during the first days of life suggests that these animals may be underweight during the fetal period but may have recovered at the end of the nutritional insult (when dams were switched to normal diet). Therefore, normal weight at birth may result from an accelerated catch-up growth, as similarly reported in animal models of maternal caloric restriction (Desai et al., 2005; Yura et al., 2005; Suzuki et al., 2010).

On the other hand, obesity in offspring exposed to maternal undernutrition has been also associated to hyperphagia (Vickers et al., 2000; Desai et al., 2005; Breton et al., 2009; Palou et al., 2010). However, our animals did not display modifications in the caloric intake relative to body weight consistent with the evidence that undernutrition in early life does not necessarily alter food intake (Yura et al., 2005; Sebert et al., 2009). Indeed, it has been reported that once offspring from animal models of maternal caloric restriction reach the same body weight as controls, their hyperphagic behavior ends (Lukaszewski et al., 2011). Under this framework, the increased body weight could be also explained by a reduction in energy expenditure linked to deregulation in endocannabinoid levels during early life, in accordance to the role of these bioactive lipids in food intake, energy balance and energy expenditure (Matias and Di Marzo, 2007; Bermudez-Silva et al., 2010).

To explain the increased adiposity found in the adult offspring from calorie-restricted dams, additional mechanisms have been proposed. For instance, inadequate nutritional conditions in early life induce upregulation of adipogenic signaling pathways, increased expression of genes involved in adipocyte differentiation (Guan et al., 2005), upregulation of lipogenic transcription factors (Desai et al., 2008) and alterations in sympathetic innervations of adipose tissue (García et al., 2011). Interestingly, the endocannabinoid system has been involved in adipogenesis and fat accumulation (Matias and Di Marzo, 2007). However, the role of the endocannabinoid system in perinatal programming has not been elucidated yet and, in the current study, we have not addressed the potential impact of maternal diet restriction on endocannabinoid signaling alterations in the periphery, such as liver, muscle or adipose tissue. However, recent preliminary data obtained using this model indicates changes in the expression of the enzymes controlling production and degradation of endocannabinoids in these peripheral tissues in adult offspring, suggesting that the observed alteration at birth might extend to the adulthood (Ramírez-López submitted). Although we did not monitor the evolution of these changes, one may speculate that endocannabinoid level alterations at birth and/or alterations in peripheral tissues involved in energy expenditure could have contributed to the increased adiposity found in adulthood. Further investigation are required to address these possibilities.

### A Moderate Maternal Caloric Restriction Increases the Propensity to Anxiety-Related Responses but does not Affect Locomotion and Food Preference in Offspring

Because of the importance of the long-term consequences of maternal undernutrition, we also explored the behavioral phenotype of adult offspring born from calorie-restricted dams. We have observed enhanced anxiety-related responses in adolescence, as measured by the elevated plus maze test, as previously described in humans (Nomura et al., 2007) and in animal models of maternal caloric restriction in early pregnancy (Erhard et al., 2004; Levay et al., 2008). Although various mechanisms have been proposed regarding the altered emotional responses following prenatal undernutrition (i.e., dysfunctions in HPA and sympathetic adrenal (Levay et al., 2010), our observation of alterations in the endocannabinoid system suggests its involvement given the predominant role played by this system in emotional control a prominent role in emotional control (Lutz, 2009; Lutz et al., 2015). In the present study, we found reduced endocannabinoid levels specifically in hypothalamus and hippocampus. Decreased levels of 2-AG in hippocampus have been associated to anxiety-related responses (Jenniches et al., 2016; Ramírez-López et al., 2016) and altered endocannabinoid signaling after cannabinoid agonist administration in early development leads to decreased emotional reactivity (Antonelli et al., 2005). Therefore, these data suggest that impairment in endocannabinoid signaling in the developing brain could lead to neurobehavioral alterations in the offspring. Nevertheless, further investigations must be pursued to establish whether decreased endocannabinoid levels at birth are correlated to the development of anxiety later in life.

## Conclusion

This is the first study to demonstrate that a moderate maternal calorie-restricted diet applied during the preconceptional and pregnancy period leads to altered endocannabinoid and NAE levels, and specifically, reduced AEA, 2-AG, AA levels in the hypothalamus and reduced AEA levels in the hippocampus. Additionally, although offspring were weaned on standard chow diet, the alterations in endocannabinoid levels may be correlated to overweight, abdominal adiposity and increased anxiety-related responses in adulthood. Therefore, these data suggest that the contribution of the endocannabinoid system in the early life programming after maternal exposure to undernutrition is relevant, especially considering that an inadequate endocannabinoid signaling could disrupt the circuitries involved in metabolism and emotional control. Further investigations aimed at understanding how and why these alterations occur might be a useful strategy in the search of efficient therapies to address impaired programming.

## Author Contributions

MTR-L, BL, RGH and FRF conceptualized and designed the experimental approaches. MTR-L, MV, FA, MA, NB, RA, DO and LO did the animal experiments, including behavioral studies and sampling. MTR-L, LB, EL and CH did the endocannabinoid measurements. JD, JS, FA and FRF did statistical analysis and graphs. JS, FRF, BL and MTR-L wrote the draft of the manuscript that was revised by all authors.

## Conflict of Interest Statement

The authors declare that the research was conducted in the absence of any commercial or financial relationships that could be construed as a potential conflict of interest.
